# Puerarin Ameliorates Caerulein-Induced Chronic Pancreatitis *via* Inhibition of MAPK Signaling Pathway

**DOI:** 10.3389/fphar.2021.686992

**Published:** 2021-06-02

**Authors:** Xiang-Peng Zeng, Jing-Hui Zeng, Xia Lin, Yan-Hong Ni, Chuan-Shen Jiang, Da-Zhou Li, Xiao-Jian He, Rong Wang, Wen Wang

**Affiliations:** Department of Digestive Diseases, 900TH Hospital of Joint Logistics Support Force, Fuzhou General Clinical Medical College of Fujian Medical University, Oriental Hospital Affiliated to Xiamen University, Fuzhou, China

**Keywords:** chronic pancreatitis, puerarin, pancreatic fibrosis, pancreatic stellate cells, caerulein

## Abstract

Pancreatic fibrosis is one of the most important pathological features of chronic pancreatitis (CP), and pancreatic stellate cells (PSCs) are considered to be the key cells. Puerarin is the most important flavonoid active component in Chinese herb *Radix Puerariae*, and it exhibited anti-fibrotic effect in various fibrous diseases recently. However, the impact and molecular mechanism of puerarin on CP and pancreatic fibrosis remain unknown. This study systematically investigated the effect of puerarin on CP and pancreatic fibrosis *in vivo* and *in vitro*. H&E staining, Sirius Red staining, qRT-PCR and Western blotting analysis of fibrosis and inflammation related genes of pancreatic tissues showed that puerarin notably ameliorated pancreatic atrophy, inflammation and fibrosis in a model of caerulein-induced murine CP. Western blotting analysis of pancreatic tissues showed the phosphorylation level of MAPK family proteins (JNK1/2, ERK1/2 and p38 MAPK) significantly increased after modeling of cerulein, while puerarin could inhibit their phosphorylation levels to a certain extent. We found that puerarin exerted a marked inhibition on the proliferation, migration and activation of PSCs, determined by CCK-8 assay, transwell migration assay, scratch wound-healing assay and expression levels of α-SMA, Fibronectin, Col1α1 and GFAP. Western blotting result demonstrated that puerarin markedly inhibited the phosphorylation of MAPK family proteins (JNK1/2, ERK1/2 and p38 MAPK) of PSCs in a dose-dependent manner whether or not stimulated by platelet-activating factor. In conclusion, the present study showed that puerarin could be a potential therapeutic candidate in the treatment of CP, and the MAPK pathway might be its important target.

## Introduction

Chronic pancreatitis (CP) is a persistent and progressive inflammatory disease, which often leads to destruction of the pancreatic parenchyma, infiltration of inflammatory cells and pancreatic calcification. Patients with CP could appear pancreatic exocrine and endocrine dysfunction, including diarrhea, steatorrhea, impaired glucose tolerance and diabetes, which seriously affect their quality of life (Beyer et al., 2020). As the most important pathological characteristic of CP, pancreatic fibrosis is the result of the imbalance between the production and degradation of extracellular matrix (ECM). The pathogenesis of pancreatic fibrosis is complex, involving multiple signaling pathways of a variety of cells, and pancreatic stellate cells (PSCs) is considered to be the key cells. Inhibiting the proliferation, migration and activation of PSCs may become a potential effective method for the treatment of CP, which is the hot spot of basic research in recent years (Ulmasov et al., 2016; Zeng et al., 2019; Che et al., 2020; Wang et al., 2020).

**FIGURE 1:**
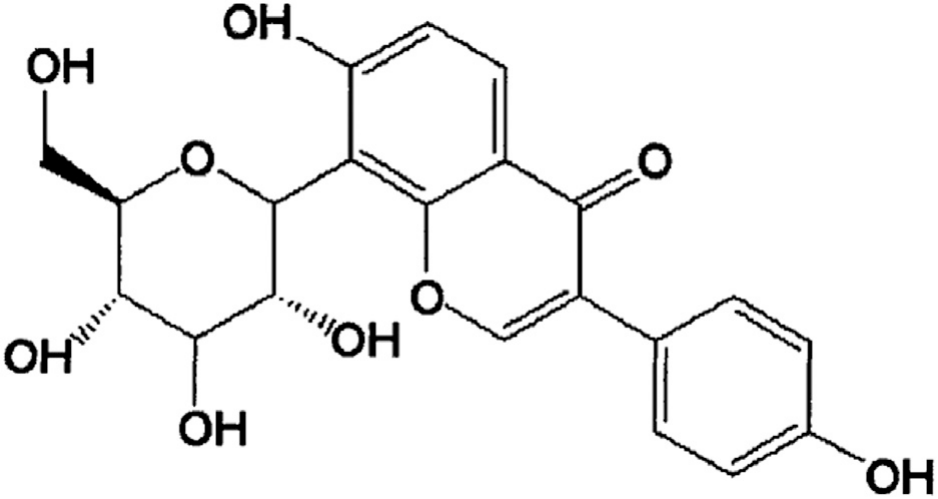
The chemical structure of puerarin.

Puerarin (chemical structure shown in Figure 1) is the most important flavonoid active component in Chinese herb *Radix Puerariae*. Puerarin has a wide range of pharmacological effects, including inhibiting platelet aggregation, anti-oxidative, anti-inflammatory, diuresis, regulating blood pressure, blood glucose and blood lipid, protecting myocardium, etc. What’s more, it has almost no toxicity. So compound preparations based on puerarin is commonly used in the adjuvant treatment of hypertension, coronary heart disease, angina pectoris, arrhythmia and other cardiovascular diseases in China (Liu et al., 2011; Chen et al., 2019; Zhou et al., 2020; Zhao et al., 2021). In recent years, it has been tolerated and approved that puerarin exhibited anti-fibrotic effect in various fibrous diseases such as liver, lung, renal and cardiac fibrosis (Zhou et al., 2017; Hu et al., 2019; Zhang et al., 2019; Li et al., 2020). However, the impact of puerarin on CP and pancreatic fibrosis remains unknown and the underlying molecular mechanism is remaining to be well investigated.

Caerulein, a gastric regulatory factor, could stimulate the secretion of stomach, bile duct and pancreas, which is similar to function of cholecystokinin. Caerulein is widely used to induce pancreatic inflammation and fibrosis in mice to establish the research model of CP. The aim of the current study was to examine the anti-fibrotic effect of puerarin in murine CP model induced by caerulein, and then investigate the effect and possible mechanism of puerarin on the proliferation, migration and activation of PSCs.

## Methods

### Animals, CP Model and Sample Analysis

A total of twenty-one C57BL/6 male mice (6–7 weeks old, 20–22 g) were purchased from SLAC Laboratory Animal Co., Ltd. (Shanghai, China), and randomly and medially divided into three groups including the control group, the caerulein (Cae) group and the Cae+Pue group. Experimental model of CP were established in mice of the Cae group and the Cae+Pue group by six hourly intra-peritoneal injections of caerulein (50 μg/kg, Sigma-Aldrich, United States), 5 days per week for a total of 6 weeks. In order to explore the therapeutic effect of puerarin, we chose to give the medicine in the second half of modeling. Upon cerulein induction, mice in the Cae+Pue group were administered with puerarin (100 mg/kg, Shandong Fangming Pharmaceutical Co., Shandong, China) dissolved in dimethyl sulfoxide by oral gavage once a day for from the first day of 4th week to the day before sacrifice (total 25 days), while mice in the Cae group were administered with vehicle solution (dimethyl sulfoxide) during the same period.

Mice were then sacrificed and analyzed five days after the last injection of caerulein. Quantitative determination of serum TGF-β1 protein level was performed using a commercial mouse TGF-β1 ELISA kit (MultiSciences, Hangzhou, China). Pancreas was rapidly removed and immediately fixed in 4% paraformaldehyde solution. Fixed tissues were sectioned and used for H&E and Sirius Red staining (Abcam, Cambridge, United Kingdom). Then the scoring method of pancreatic tissue of CP referred to Westerloo standard (van Westerloo et al., 2005), including pathological changes, acinar atrophy, fibrosis and inflammatory cell infiltration. The total pancreatic pathological score range is 0–14 points, and the higher the score, the more severe the CP was. The positive area of Sirius Red staining was calculated and compared by Image J software. Ethical approval for the handling of mice and experimental procedures had been obtained from the Animal Care Committee of 900TH Hospital of Joint Logistics Support Force.

### Cell culture

Human pancreatic stellate cells (HPSCs) were obtained from Prof. Logsdon CD (Department of Cancer Biology, University of Texas MD Anderson Cancer Centre, Houston, Texas, United States), which were isolated using the outgrowth method from pancreatic adenocarcinoma samples after surgical resection. All cells were incubated at 37°C in 5% CO_2_ and seeded in Dulbecco’s modified eagle medium (Hyclone, Logan, United States), supplemented with 1% penicillin/streptomycin (Gibco, California, United States) and 15% fetal bovine serum (Gibco, California, United States). For pharmacologic efficacy analysis and mechanistic study, HPSCs were treated with different concentrations of puerarin or control dimethyl sulfoxide or platelet-activating factor (PAF) after reaching 70% confluence. Cells were harvested at various time points and then subjected to quantitative real-time PCR (qRT-PCR) or Western blotting as described below.

### Quantitative Real-Time PCR

Total RNA was extracted from cells or pancreas of mice using Trizol reagent (Invitrogen, California, United States), and then 1 μg of RNA was reverse transcribed into cDNA using the RevertAid First Strand cDNA Synthesis Kit (Thermo Scientific, Waltham, United States). Then qRT-PCR was carried out in triplicate with Hieff™ qPCR SYBR^®^ Green Master Mix (YEASEN Biotechnology, Shanghai, China) on a LightCycler^®^ 480 II System (Roche, Sandhofer, Germany). The primer sequences were listed in Table 1.

**TABLE 1:** Primer Sequences Used for qRT-PCR Analysis.

Gene		Primer sequence (5′→3′)
Murine α-SMA	Forward	GCC​AGT​CGC​TGT​CAG​GAA​CCC
	Reverse	CCA​GCG​AAG​CCG​GCC​TTA​CA
Murine Col1α1	Forward	CGCCATCAAGGTCTACTG
	Reverse	ACGGGAATCCATCGGTC
Murine IL-6	Forward	AGT​TGC​CTT​CTT​GGG​ACT​GA
	Reverse	TCC​ACG​ATT​TCC​CAG​AGA​AC
Murine TNF-α	Forward	CCA​AAG​GGA​TGA​GAA​GTT​CC
	Reverse	CTC​CAC​TTG​GTG​GTT​TGC​TA
Murine GAPDH	Forward	GGT​CGG​TGT​GAA​CGG​ATT​TG
	Reverse	TGT​AGA​CCA​TGT​AGT​TGA​GGT​CA
Human α-SMA	Forward	CCG​ACC​GAA​TGC​AGA​AGG​A
	Reverse	ACA​GAG​TAT​TTG​CGC​TCC​GAA
Human Col1α1	Forward	CAG​CCG​CTT​CAC​CTA​CAG​C
	Reverse	TTT​TGT​ATT​CAA​TCA​CTG​TCT​TGC​C
Human GAPDH	Forward	GAC​AGT​CAG​CCG​CAT​CTT​CT
	Reverse	GCG​CCC​AAT​ACG​ACC​AAA​TC

### Cell Viability Assay

Cells viability was detected by the Cell Counting Kit-8 (CCK-8, KeyGEN, Shanghai, China) according to the manufacturer’s instructions. The well-growing HPSCs were collected and inoculated into a 96-well plate at a density of 2,000 cells/well and cultured in 5% CO_2_ environment at 37°C. These cells were added 10 μl/well of test solution after indicated treatments, and then incubated for 3 h at 37°C. The absorbance at 450 nm was measured using a microplate reader. Results were obtained from three independent experiments in triplicate.

### Western Blotting

Cell and pancreatic tissue lysates were prepared using RIPA lysis buffer (Beyotime Biotechnology, Shanghai, China) and purified by centrifugalization and separated by 10% SDS-PAGE, subsequently transferred to PVDF membrane (Merck Millipore, Darmstadt, Germany). After blocking with 5% skimmed milk for 1 h, the PVDF membranes were then incubated with various primary antibodies at 4°C overnight, followed by incubation with horseradish peroxidase-conjugated secondary antibodies (Jackson ImmunoResearch, West Grove, United States) for 1.5 h at room temperature. The target proteins were then visualized using the Amersham Imager 600 ECL system (GE, Fairfield, United States). GAPDH (CMCTAG, WI, United States) was used as internal control. Primary antibodies used in this study were described in Table 2.

**TABLE 2:** Primary antibody used for Western blotting analysis.

Antibody	Company	Dilution
PCNA	Cell Signaling Technology (#13110)	1:1,000
α-SMA	Cell Signaling Technology (#19245)	1:1,000
GFAP	Cell Signaling Technology (#12389)	1:1,000
Fibronectin	Santa Cruz Biotechnology (sc-8422)	1:1,000
JNK1/2	Cell Signaling Technology (#9252)	1:1,000
p-JNK1/2	Cell Signaling Technology (#4668)	1:1,000
ERK1/2	Cell Signaling Technology (#4695)	1:1,000
p-ERK1/2	Cell Signaling Technology (#4370)	1:2,000
p38 MAPK	CALBIOCHEM (#506123)	1:1,000
p-p38 MAPK	Cell Signaling Technology (#4511)	1:1,000

### Cell Migration Function

The cell invasion activity was analyzed using a Falcon^®^ Cell Culture Inserts (Corning, NY, United States). HPSCs were seeded into the insert at a density of 1 × 10^4^/well with 100 μL serum free culture medium supplemented with puerarin (50 or 100 nM) or control dimethyl sulfoxide, and 500 μl culture medium supplemented with 10% fetal bovine serum were added into the lower 12-well plates. After incubation at 37°C for 24 h, cells migrating through the transparent polyethylene terephthalate membrane (8.0 μm) were stained with crystal violet, and then the cell images were captured and quantified.

Wound-healing Assay was another important way to evaluate migration function of cells. About 5 × 10^5^ cells/well were seeded in the 6-well plates at 37°C. When cells adhered to the plate, original culture medium was discarded. After phosphate buffer saline washes once, cells were incubated with serum-free culture medium for 24 h. Then, the confluent cell monolayer was scratched with a sterile pipette tip (200 μl) to create a narrow wound-like gap. Phosphate buffer saline wash once for removing non-adherent cells, and then serum-free culture medium supplemented with puerarin (100 nM) or control dimethyl sulfoxide were added for further culture. The wound healing process was monitored at 0, 6 and 12 h using an inverted microscope.

### Statistical Analysis

Kolmogorov-Smirnov test was used to check the normality of continuous variables, and normal distribution data was presented as mean ± SD, and then data were analyzed and compared by unpaired Student’s t-test or one-way ANOVA followed by Tukey’s multiple comparison tests using the GraphPad Prism software (Version 5.01). Non-parametric statistical tests were performed for comparisonamong non-normal distribution of data. Statistical analyses were conducted at a significance level of *p* value <0.05 for all analyses.

## Results

### Puerarin Ameliorated Caerulein-Induced Pancreatic Inflammation and Fibrosis *In Vivo*

**FIGURE 2:**
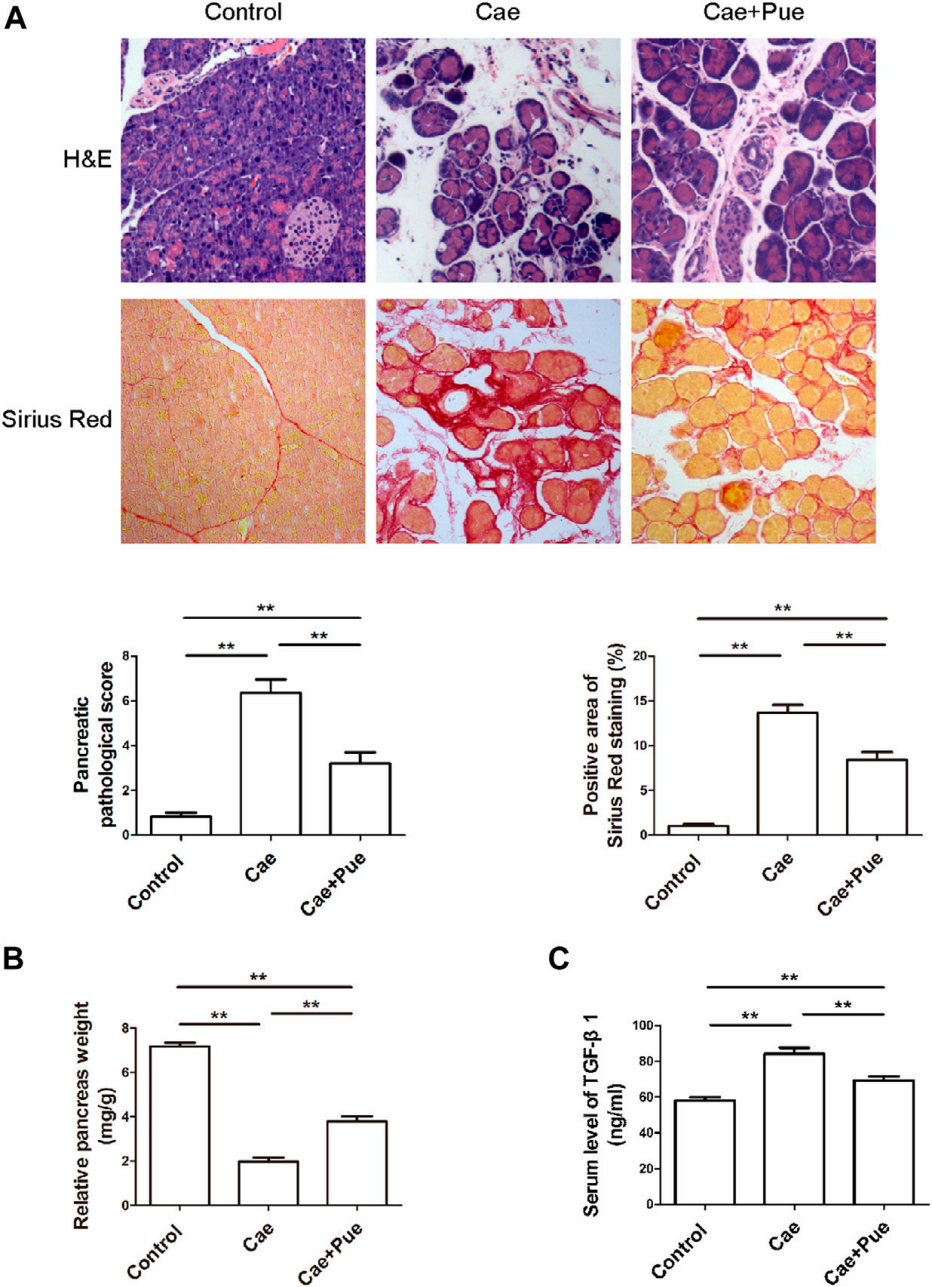
Puerarin ameliorated caerulein-induced pancreatic inflammation and fibrosis *in vivo*. **(A)** H&E and Sirius Red staining of paraffin-embedded pancreatic sections from each group. **(B)** Relative pancreas weights were calculated and compared among three groups. **(C)** Serum TGF-β1 level was measured by ELISA and compared among three groups.

Experimental CP model was induced in C57BL/6 male mice by repeated intra-peritoneal injections of caerulein for 6 weeks. Mice in the Cae+Pue group were orally administered with puerarin (100 mg/kg) once a day during second half of caerulein injection. H&E staining and Sirius Red staining of pancreatic tissues were performed to evaluate its anti-fibrotic and anti-inflammatory effects (Figure 2A). Compared with the Cae group, the major characteristics of CP (including the atrophy of acinar cells, infiltration of inflammatory cells, accumulation of interstitial ECM proteins and so on), the pancreatic pathological score and positive area of Sirius Red staining were all markedly attenuated in the Cae+Pue group. As illustrated in Figure 2B, the relative pancreas weight of the Cae group was notably lower than that of control group (*p* < 0.05). Whereas in the Cae+Pue group, the relative pancreas weight were partially improved (*p* < 0.05), indicating that repetitive injections of caerulein could lead to atrophy of the pancreas, and puerarin administration could mitigated this effect. Concomitantly, serum TGF-β1 levels were significantly increased in the Cae group due to repeated caerulein injections, while the level was markedly ameliorated by additional puerarin administration (Figure 2C).

**FIGURE 3:**
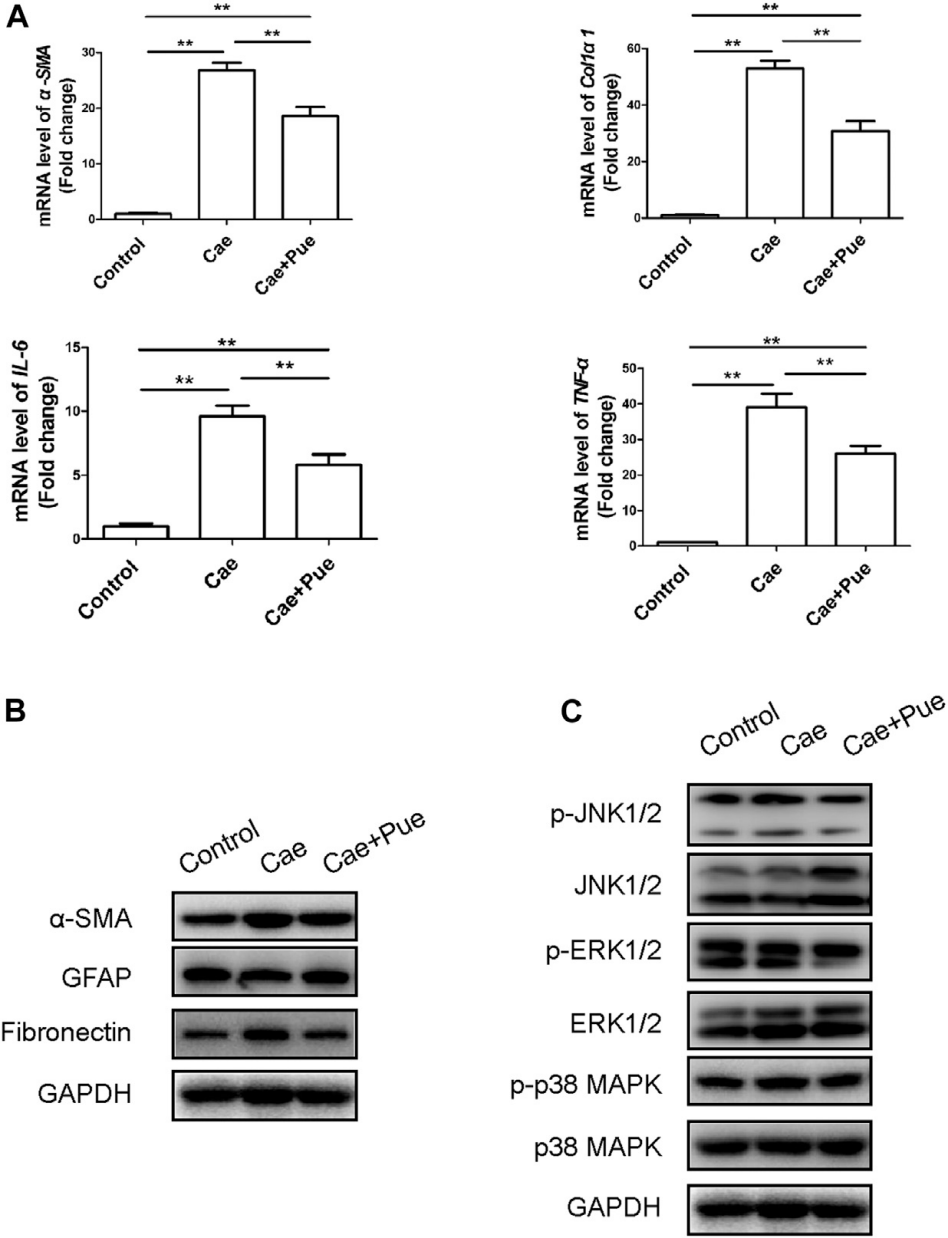
Puerarin ameliorated pancreatic inflammation and fibrosis *in vivo* maybe through inhibiting the phosphorylation activation of MAPK family proteins. **(A)** Expression of fibrotic and inflammatory associated genes (α-SMA, Col1α1, TNF-α and IL-6) of pancreatic tissues were quantified by qRT-PCR and compared among three groups. Data were presented as mean ± SD of at least three independent experiments. ^*^ *p* < 0.05, ^**^ *p* < 0.01. **(B)** The protein expression levels of fibrotic and inflammatory associated genes (α-SMA, GFAP and Fibronectin) of pancreatic tissues were tested by Western blotting analysis and compared among three groups. **(C) **The total proteins and phosphorylation proteins expression levels of MAPK family proteins (including JNK1/2, ERK1/2 and p38 MAPK) of pancreatic tissues were tested by Western blotting analysis and compared among three groups.

In addition, qRT-PCR and Western blotting results further confirmed that puerarin notably reduced the expression levels of fibrosis and inflammation related genes including α-Smooth muscle actin (α-SMA), Col1α1, Fibronectin, TNF-α and IL-6, while increased the protein level of glial fibrillary acidic protein (GFAP) in pancreatic tissues (Figures 3A,B). We also made a preliminary analysis on the mechanism of puerarin’s treatment on pancreatic fibrosis and inflammation through Western blotting experiment. As illustrated in Figure 3C, the phosphorylation level of MAPK family proteins (including JNK1/2, ERK1/2 and p38 MAPK) significantly increased after modeling of cerulein, while puerarin could inhibit their phosphorylation levels to a certain extent. Taken together, these data suggested that 6-weeks intra-peritoneal injections of caerulein could achieve animal modeling of CP, and puerarin administration could significantly meliorate the pancreatic inflammation and fibrosis *in vivo* maybe through inhibiting the phosphorylation of MAPK family proteins.

### Puerarin Inhibited the Proliferation of HPSCs

**FIGURE 4:**
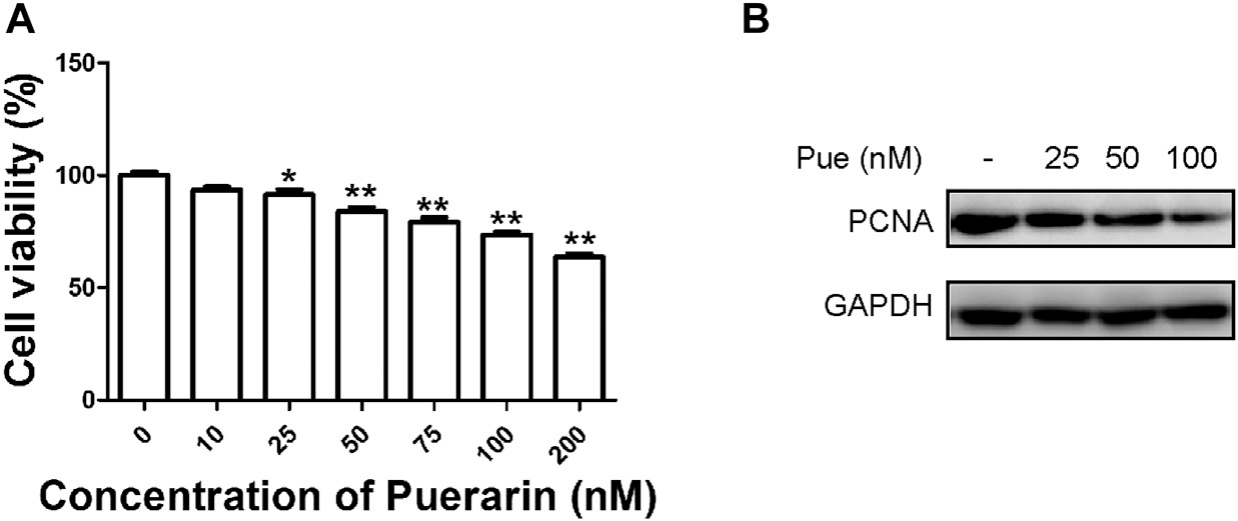
Puerarin inhibited the proliferation of HPSCs. **(A)** Cell viability of HPSCs was tested by Cell Counting Kit-8 assay 48 h after treatment with puerarin. ^*^ *p* < 0.05, ^**^ *p* < 0.01. **(B)** The protein expression levels of PCNA were tested in HPSCs after treatment with different doses of puerarin (25, 50, 100 nM) for 24 h by Western blotting analysis.

As shown in Figure 4A, the cell viability of HPSCs gradually decreased with increasing dose of puerarin administrated. In consideration of the possible cytotoxicity of puerarin, we chose 100 nM (relevant cell viability was about 80%) as the maximum dose in following studies. To validate the above findings, we next performed Western blotting on HPSCs by co-administration of puerarin (Figure 4B). The protein level of cell proliferation marker PCNA decreased with increasing dose of puerarin treatment, suggesting that puerarin treatment dose-dependently inhibited the proliferation of HPSCs.

### Puerarin Inhibited the Migration of HPSCs

**FIGURE 5:**
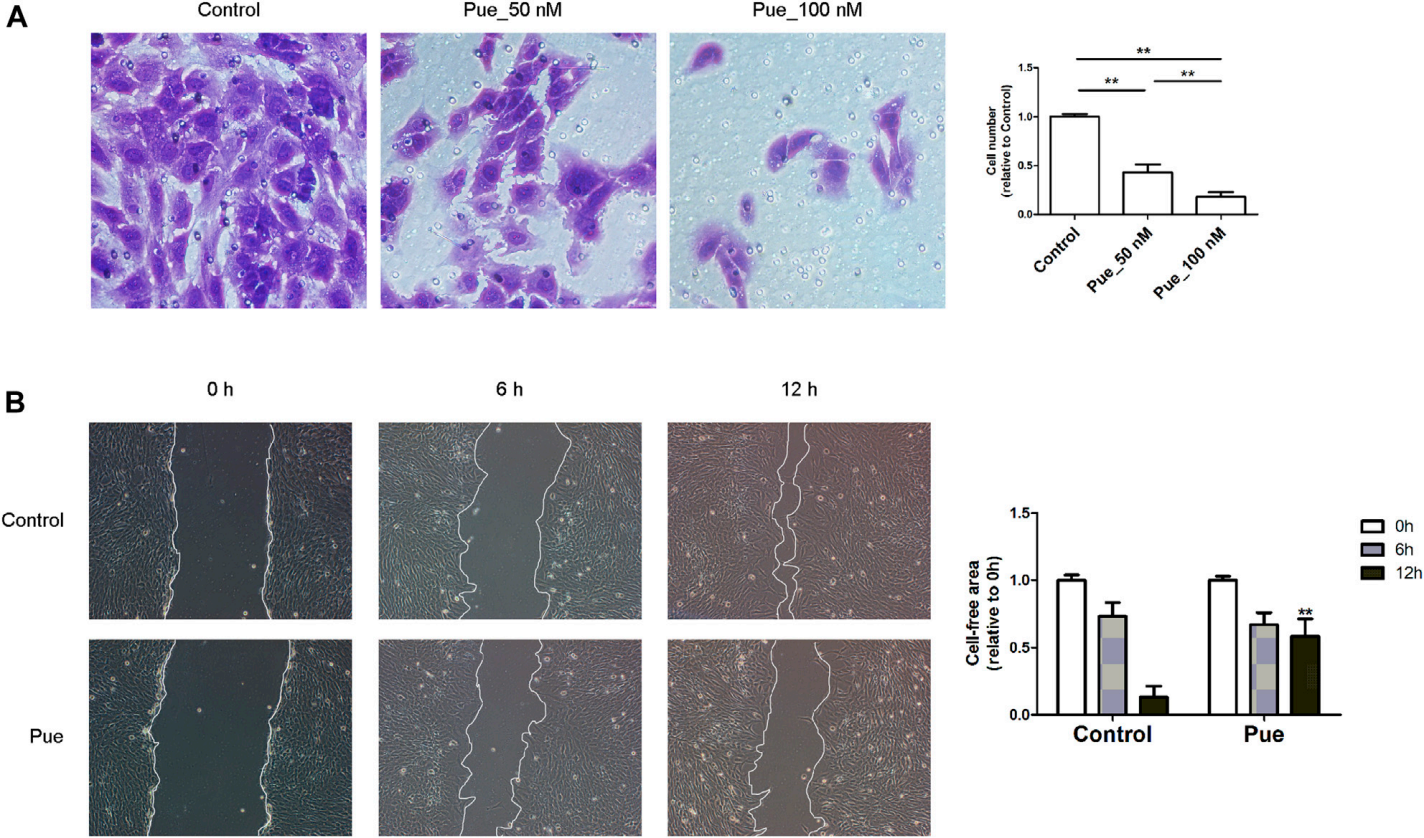
Puerarin inhibited the migration of HPSCs. **(A)** Transwell cell migration assay result in HPSCs. Images showing cells that migrating through the transparent polyethylene terephthalate membrane were captured after treatment with or without puerarin (50, 100 nM) for 24 h. The average cell numbers of six random areas of each group were calculated and presented in the bar chart. ^*^ *p* < 0.05, ^**^ *p* < 0.01. **(B)** Wound-healing assay result in HPSCs treated with or without puerarin (100 nM), images were captured at 0, 6 and 12 h after scratching. Cell-free area was quantified using Image J and statistically presented in the histogram. ^*^ *p* < 0.05, ^**^ *p* < 0.01 vs. control.

The migratory capacity is another important biological aspect of PSCs that closely related to CP, which can be measured by transwell migration assay and scratch wound-healing assay. Transwell migration assay result showed that puerarin dose-dependently reduced the number of HPSCs migrated through the transparent polyethylene terephthalate membrane (Figure 5A). As scratch wound-healing assay result shown in Figure 3B, the velocity of HPSCs migrating into the wound gap area markedly decreased under treatment with puerarin (Figure 5B), further confirming that puerarin markedly inhibited the migration of HPSCs.

### Puerarin Inhibited the Activation of HPSCs

**FIGURE 6:**
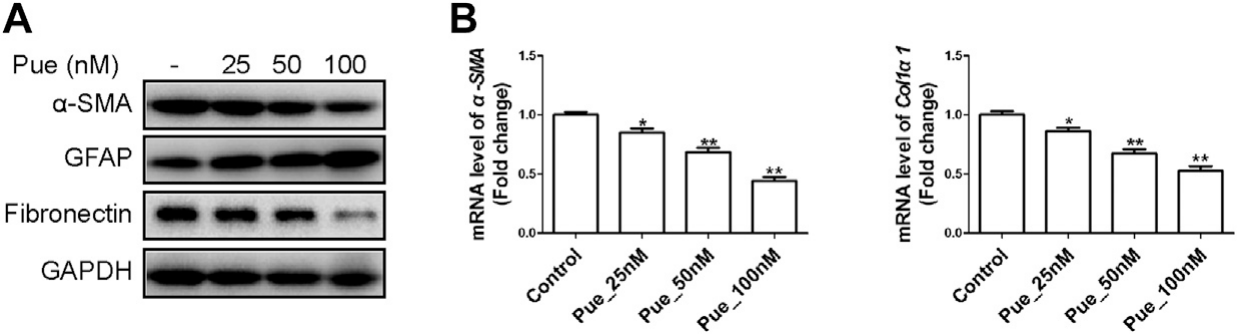
Puerarin inhibited the activation of HPSCs. **(A)** The protein expression levels of α-SMA, GFAP and Fibronectin were tested in HPSCs after treatment with different doses of puerarin (25, 50, 100 nM) for 24 h by Western blotting analysis. **(B)** The mRNA expression levels of α-SMA and Col1α1 were tested in HPSCs after treatment with different doses of puerarin (25, 50, 100 nM) for 24 h by qRT-PCR analysis. ^*^ *p* < 0.05, ^**^ *p* < 0.01 vs. control.

Pancreatic fibrosis in CP is closely related to cell activation of PSCs. As shown in Figure 6, results from Western blotting and qRT-PCR assays illustrated that puerarin administration dose-dependently decreased the expression of these fibrogenic genes including α-SMA, Fibronectin and Col1α1, while increased the protein level of GFAP in a dose-dependent manner. Therefore, puerarin treatment could markedly mitigate the activation of HPSCs.

### Puerarin Suppressed the Activity of MAPK Cascades in HPSCs

**FIGURE 7:**
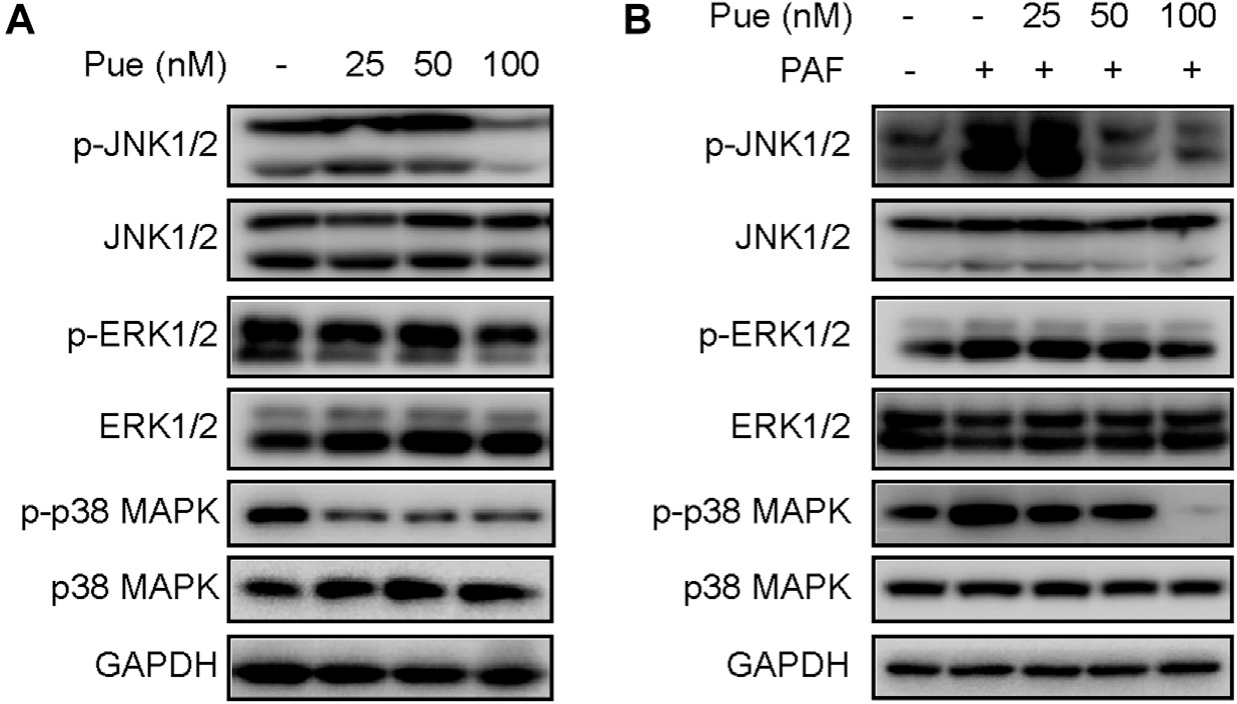
Puerarin suppressed the activity of MAPK cascades in HPSCs. **(A)** The total proteins and phosphorylation proteins expression levels of MAPK family proteins (including JNK1/2, ERK1/2 and p38 MAPK) were tested in HPSCs after treatment with different doses of puerarin (25, 50, 100 nM) for 24 h by Western blotting analysis. **(B)** The total proteins and phosphorylation proteins expression levels of MAPK family proteins were tested in HPSCs after stimulation of PAF (10 nM) and treatment with different doses of puerarin (25, 50, 100 nM) for 24 h by Western blotting analysis.

To further investigate the mechanism of puerarin on pancreatic fibrosis, we detected the expression levels of total proteins and phosphorylation proteins of MAPK family proteins (including JNK1/2, ERK1/2 and p38 MAPK) using Western blotting experiments in HPSCs. As shown in Figure 7, whether or not stimulated by PAF, puerarin could notably inhibit the phosphorylation of JNK1/2, ERK1/2 and p38 MAPK in a dose-dependent manner, but did not significantly affect their expression levels of total proteins.

## Discussion

To our knowledge, the current study is the first study to investigate the treatment effect of puerarin on caerulein-induced murine CP, and the result illustrated that puerarin could ameliorate the pancreatic inflammation and fibrosis by reducing proliferation, migration and activation of PSCs. For its mechanism, we found that puerarin could notably inhibited the phosphorylation of MAPK family proteins (JNK1/2, ERK1/2 and p38 MAPK) in a dose-dependent manner. Taken together, puerarin exerted obvious anti-fibrotic and anti-inflammatory effects on CP and may serve as a potential therapeutic agent in clinical setting.

At present, it is recognized that repeating intra-peritoneal injection of caerulein could significantly induce pancreatic inflammation and fibrosis in mice, so it has become one of the most commonly used animal models of CP (Xue et al., 2016; Mukherjee et al., 2020; Ren et al., 2020; Tao et al., 2020). In the current study, H&E and Sirius staining revealed a marked increase in number of atrophic acinar cells and infiltrating inflammatory cells, with a markedly increased deposition of collagen fibers in the Cae group, as well as pancreatic fibrosis were directly observed. Furthermore, compared with the control group, the relative pancreas weight in the Cae group was significantly decreased while serum TGF-β1 level was significantly increased. These results suggest that the CP *in vivo* model was successfully established through caerulein in mice.

Puerarin is a monomer compound extracted and isolated from dried *Radix Puerariae*, which has been exhibited favorable anti-inflammatory and anti-fibrotic effect for multiple organs recently (Huang et al., 2018; Ni et al., 2020). Lin *et al.* reported that ischemia-reperfusion injury could induce uterine fibrosis, and puerarin exerted an improvement effect on uterine fibrosis by downregulating the activity of mast cell chymase, TGF-β, α-SMA, and the Wnt/β-catenin pathway (Lin et al., 2017). Jin *et al.* revealed that puerarin protected against cardiac fibrosis induced from transverse aorta constriction, which may be attributed to the upregulation of PPAR-γ and the inhibition of TGF-1/Smad2-mediated endothelial-to-mesenchymal transition (Jin et al., 2017). The current study illustrated that pancreatic atrophy, inflammation, fibrosis, expression levels of α-SMA (marker of PSCs activation), Col1α1 (major fibrotic marker), TNF-α and IL-6 (inflammatory markers) were significantly alleviated in the Cae+Pue group compared with the Cae group. These results suggested that puerarin exhibited an anti-fibrotic effect in CP, but the underlying mechanism remains unknown.

PSCs are widely regarded as the key fibrogenic cell that coordinates pancreatic ECM formation. Activated PSCs secrete abundant ECM proteins including collagens, α-SMA, fibronectin and desmin for tissue repair and kinds of cytokines and chemokines that would facilitate the infiltration of inflammatory cells. If injury factors and inflammatory responses remain unresolved, the persistent activation of PSCs would promote a state of chronic fibro-inflammation and development of pancreatic fibrosis. Therefore, inhibiting PSCs activation is one of promising therapeutic approach for reversal of pancreatic fibrosis and CP. Considering the anti-proliferative property of puerarin as reported in previous studies (Wan et al., 2018; Li et al., 2020), we first tested its effect on the proliferation of cultured HPSCs. CCK-8 and Western blotting results showed the cell viability and PCNA level of HPSCs gradually decreased with increasing dose of puerarin administrated, suggesting that puerarin treatment inhibited the proliferation of HPSCs in a dose-dependent manner. Scratch wound-healing assay and transwell migration assay results indicated that puerarin dose-dependently inhibited the migration of HPSCs. Pancreatic fibrosis in CP is closely related to the activation of PSCs. We found that puerarin treatment could significantly decrease the expression of α-SMA, Fibronectin and Col1α1 and increased the expression of GFAP (a marker of quiescent PSCs) in a dose-dependent manner through qRT-PCR or Western blotting. So puerarin treatment could markedly mitigate the activation of HPSCs.

We further investigated downstream key pathway that response to puerarin treatment for pancreatic fibrosis. MAPK family proteins (including JNK1/2, ERK1/2 and p38 MAPK) is a highly conserved serine protein kinase found in cytoplasm, and phosphorylation of MAPK plays an essential role in fibrosis of multiple organs (Peng et al., 2019; Geng et al., 2020; Guo et al., 2021). Several recent studies suggested that MAPK cascades participated in the regulation of PSCs activation and progress of pancreatic fibrosis (Xia et al., 2018; Xu et al., 2018; Zeng et al., 2019; An et al., 2020). Xu *et al.* demonstrated that ERK and JNK were directly involved in the activation of PSCs induced by TGF-b1 and the development of pancreatic fibrosis (Xu et al., 2018). It is worth noting that several studies have shown that regulating MAPK pathway may be one of important mechanisms for the anti-fibrotic effect of puerarin (Zhou et al., 2017; Cai et al., 2018; Li et al., 2019). Li *et al.* found that puerarin may reduce activation hepatic stellate cell and alleviate extracellular matrix protein expression levels by inhibiting the TGF-β/ERK1/2 pathway in liver fibrosis (Li et al., 2019). Zhou *et al.* reported that puerarin could ameliorate renal fibrosis by inhibiting oxidative stress induced-epithelial cell apoptosis through MAPK signaling (Zhou et al., 2017). Cai *et al.* revealed that puerarin could prevent cardiac fibrosis *via* activation of Nrf2 and inactivation of p38-MAPK (Cai et al., 2018). Our study also demonstrated that puerarin markedly inhibited the phosphorylation of JNK1/2, ERK1/2 and p38 MAPK of HPSCs in a dose-dependent manner whether or not stimulated by PAF, which suggests that the anti-fibrotic effect of puerarin in pancreatic fibrosis may occur *via* MAPK signaling pathway. However, whether puerarin binds to other functional domains of MAPK family proteins requires further investigation.

Of course, there are still several limitations exist in this study. Firstly, although cell culture of PSCs is considered as a common model for *in vitro* study of pancreatic fibrosis, it is hard currently to confirm direct clinical relevance of our findings. Secondly, the detailed mechanism of puerarin on the proliferation, migration and activation of PSCs were not fully elucidated, which may be key directions for future research. Thirdly, only one single model (caerulein-induced mouse CP) was used in this study.

In conclusion, the present study systematically investigated the mitigative effect of puerarin on pancreatic inflammation and fibrosis *in vivo* and *in vitro*. Mechanistically, puerarin exerted a marked suppressive effect on the proliferation, migration, activation of PSCs and the phosphorylation of JNK1/2, ERK1/2 and p38 MAPK. These findings suggested that puerarin could be a potential therapeutic candidate in the treatment of CP, and the MAPK pathway might be its important target.

## Data Availability

The raw data supporting the conclusions of this article will be made available by the authors, without undue reservation, to any qualified researcher.

## Abbreviations

CP, chronic pancreatitis; ECM, extracellular matrix; PSCs, pancreatic stellate cells; α-SMA, α-Smooth muscle actin; GFAP, Glial fibrillary acidic protein.
